# Tannic Acid and Ca^2+^ Double-Crosslinked Alginate Films for Passion Fruit Preservation

**DOI:** 10.3390/foods12213936

**Published:** 2023-10-27

**Authors:** Jun Yang, Tao Fei, Wanli Zhang, Xinli Cong

**Affiliations:** 1 School of Life Sciences, Hainan University, Haikou 570228, China; yjun0893@gmail.com; 2 School of Food Science and Engineering, Hainan University, Haikou 570228, China; feitao_fse@hainanu.edu.cn (T.F.); zwl@hainanu.edu.cn (W.Z.)

**Keywords:** alginate, tannic acid, crosslinking, film properties, fruit coating

## Abstract

In this study, the interaction of different concentrations of tannic acid (TA) (10%, 20%, and 30% *w*/*w*) and Ca^2+^ with alginate (SA) was utilized to create double-crosslinked SA films. The resulting films were evaluated for their optical, mechanical, water resistance, and barrier properties, and their microstructure and intermolecular interactions were also characterized. The SA films containing 20% TA showed the best mechanical properties, with an observed increase in tensile strength of 22.54%. In terms of water vapor permeability, the SA film containing 30% TA exhibited the highest barrier property, which was 25.36% higher than that of the pure SA film. Moreover, TA demonstrated a strong UV absorption ability, resulting in a nearly 0% UV transmittance of the SA film at 280 nm. It can be seen that SA films containing 20% TA have excellent barrier and mechanical properties, and the development of such films will be applied to the storage and packaging of fresh food. It is worth noting that this work also investigated the effect of SA coatings containing different concentrations of TA on the preservation of passion fruits for 7 days. The results revealed that passion fruits treated with SA coatings containing a 30% TA concentration maintained a better appearance on the 7th day and had the lowest weight loss and crumpling indices of approximately 8.98% and 2.17, respectively, compared to the other treatment groups. Therefore, based on the overall results, the addition of 30% TA to SA coatings proved to be more effective and can be considered a promising approach for delaying fruit senescence and decay.

## 1. Introduction

The primary role of food packaging in the processing sector is to protect products from microbial decay, physical damage, chemical breakdown, ultraviolet radiation, and pollutants. Traditional packaging materials, mainly derived from synthetic plastics, have led to detrimental ecological consequences such as soil and water contamination and harm to marine life [1]. Microplastics, which are plastic particles smaller than 5 mm, have been detected in various environments, including air, water, food, and animal digestive systems. Recent studies have even found microplastics in human blood, possibly due to exposure to plastic-contaminated water and marine-based food sources [2]. To address these concerns, biopolymers, known for their eco-friendly, biodegradable, biocompatible, and non-toxic properties, offer a promising alternative to conventional plastics [1,3]. The use of natural polymers such as polysaccharides (cellulose, sodium alginate (SA), chitosan, starch, pectin, gums, and dextrin), proteins (gelatin, gliadin, zein, seitan, casein, whey, and soybean), and lipids (fatty acids and wax) for creating biodegradable packaging materials from renewable sources has gained significant attention in the food packaging industry [4,5]. Coatings and edible packaging films, with their protective and inhibitory properties against food degradation, can effectively safeguard food items from physical, chemical, and microbial spoilage during storage [6].

SA, a structural polysaccharide derived from brown seaweed, is composed of glucuronic (G) and mannuronic (M) acid units, which form various regions, including M-blocks, G-blocks, and alternating sequences (MG-blocks). The distribution of these sequential arrangements depends on the specific source of SA [7]. SA, obtained commercially from seaweed, finds diverse applications in fields such as food packaging, tissue engineering, biomedicine, and pharmaceuticals thanks to its non-toxic, biodegradable, biocompatible nature and its ability to form gels [8]. However, SA films face limitations such as fragility, high water sensitivity, limited thermal stability, and inadequate gas barrier properties, which hinder their wider implementation [9]. One of the most valuable characteristics of alginates is their ability to interact with polyvalent metal cations, particularly calcium ions, resulting in the formation of robust gels or insoluble polymers. Divalent ionic crosslinking is the most effective method for producing insoluble SA films, as numerous studies have shown that the interaction with polyvalent metal cations can enhance the water resistance and mechanical properties of SA-based films [10].

Currently, the main technique for creating water-resistant SA films involves external crosslinking, as internal crosslinking with cations leads to rapid gelation of SA and hinders film casting. However, external crosslinking with calcium ions results in fast crosslinking and high swelling, leading to film folding and inhomogeneity [11]. To address this issue, alternative crosslinking agents can be employed to enhance the properties of SA films by forming multi-network crosslinks. Tannic acid, a natural phenolic acid known for its antibacterial and antioxidant properties, is widely used in the food industry [12]. As a natural crosslinking agent, tannic acid has been shown to improve the performance of various biopolymer-based food packaging films, including casein, gelatin, chitosan, and polyvinyl alcohol [13,14,15,16]. Recent studies have also demonstrated the interaction between tannic acid and SA molecular chains through hydrogen bonding, leading to improved SA film properties [2,17].

However, the existing studies mainly focus on the direct crosslinking of SA with tannic acid. Limited information is available on the impact of tannic acid and calcium ion crosslinking on the properties of SA films. Therefore, this study aims to investigate the effects of different tannic acid crosslinking concentrations on the properties of SA/Ca^2+^ films. Additionally, we characterized the composite SA/Ca^2+^ films using scanning electron microscopy, Fourier transform infrared spectroscopy, X-ray diffraction, and thermogravimetric analysis to analyze their microstructure, intermolecular forces, and thermal stability. Importantly, SA films containing TA can be used to preserve fresh food and can significantly extend the storage and preservation time of fresh food.

## 2. Materials and Methods

### 2.1. Materials

Pharmaceutical-grade sodium alginate (SA, AR) with a low viscosity and a purity of >74% was obtained from Zhengzhou Feynman Biotechnology Co. (Zhengzhou, China). Meanwhile, we determined the molecular weight of sodium alginate by GPC gel permeation chromatography (Agilent RID G1362A, Agilent, Santa Clara, CA, USA), which was 439.735 KDa. Tannic acid (TA) used in the study was brownish-yellow in color and was purchased from Tianjin Beichen Founder Reagent Factory (Tianjin, China). Other reagents were obtained from our laboratory and were of analytical grade. The fruits were purchased from Baoting Hesheng Agricultural Development Co., Ltd. (Sanya, Hainan, China), were uniform in size, free of any physical damage, and arrived at our laboratory in Sanya, Hainan, China, within 24 h of picking.

### 2.2. Preparation of Different Concentrations of SA Films

SA films were fabricated using a solution casting method [18]. The 3% (*w*/*v*) SA solution was prepared by dissolving SA powder in distilled water. Subsequently, TA solution in water was added to the 3% (*w*/*v*) SA solution, obtaining a mixed film-forming solution with a SA concentration of 2% (*w*/*v*) and TA concentrations of 10%, 20%, and 30% (*w*/*w* based on SA solid mass). Glycerol (30% *w*/*w* based on SA solid mass) was then added to the mixture as a plasticizer. The resulting mixed film-forming solution was poured into circular polyethylene Petri dishes and dried in an oven at 60 °C for 5 h to obtain fully dried SA-TA composite films, designated as SA/TA10, SA/TA20, and SA/TA30 films. All films were cross-linked with calcium cations by immersing the SA/TA films in a 50% (*v*/*v*) ethanol–water solution containing 2% (*w*/*v*) calcium chloride for 2 min. Finally, the films were conditioned at a relative humidity (RH) above 60% and at room temperature for 72 h.

### 2.3. Thickness, Color, and Optical Properties of Film

Film thickness was measured with a digital micrometer (DL9325, Ningbo Deli Tool Co., Ltd., Ningbo, Zhejiang, China) with an accuracy of 0.01 mm. The color parameters L* (Brightness), a* (Redness/Greenness), and b* (Yellow/Blue Degree) of the films were evaluated with a reflectance spectrophotometer (TA-XT plus 40762, Konica minolta sensing, Inc, Tokyo, Japan). The transparency of the films was calculated using our previously established method [19]. The transparency of the film is reflected by the calculation of opacity; the greater the opacity, the worse the transparency of the sample. The opacity equation of the film is shown in (1):

(1) $$\begin{array}{l} p=\frac{A}{m} \end{array}$$

Herein, p represents the opacity of the SA film sample (%), *A* is the absorbance of films at 600 nm, and *m* is the thickness of films (mm).

### 2.4. Characterization of Films

Before performing SEM scanning electron microscopy, we cut SA films containing different concentrations of tannic acid into rectangles of appropriate size and sprayed gold on the surface. In contrast, in the cross-sectional analysis of films, the films were fractured in liquid nitrogen. Subsequently, the microstructure of the SA films was observed using a scanning electron microscope (SEM) (SEM, Zeiss GeminiSEM 300, Jena, Germany) at magnifications of 3000× and 1000× and an accelerating voltage of 20 kV. Samples were analyzed for chemical bonds and functional groups using an FTIR spectrometer (Thermo Scientific iN10, Thermo Fisher Scientific, Waltham, MA, USA) in the wavelength range of 4000–400 cm^−1^ with a resolution of 4 cm^−1^. The X-ray diffraction (XRD) spectrum of the film samples was obtained using a SmartLab SE X-ray diffractometer (Rigaku SmartLab SE, Rigaku Co., Akishima, Japan) with a diffraction angle ranging from 10° to 80° and a scanning rate of 2°/min. The UV and visible light transmittance of the samples were measured using a UV-5500PC spectrophotometer (UV-5500PC, Yuananalysis Ltd., Shanghai, China) in the wavelength range of 200–800 nm. The thermal stability of the films was assessed using a TGA 550 thermogravimetric analyzer (Discovery TGA550, New Castle, TA, USA) in the temperature range of 40–400 °C. The heating rate was set at 10 °C/min under a nitrogen atmosphere with a flow rate of 50 mL/min.

### 2.5. Moisture Content and Water Solubility

The moisture content and water solubility of the SA films were determined using previously established methods [18]. The film was dried at 105 °C until a constant weight was achieved. Subsequently, the film was immersed in distilled water and agitated at room temperature for 24 h. Afterward, the film was dried again at 105 °C until a constant weight was obtained. The mass difference was used to calculate the moisture content and water solubility of the film.

### 2.6. Water Vapor Permeability and Water Contact Angle

The water vapor permeability (WVP) of the film was determined following a previously established method [19]. A film sample was sealed over a weighing cup with dimensions of 3 cm in diameter and 8 cm in depth, which contained 10 g of CaCl_2_. The cup was then placed in a desiccator with saturated potassium iodide at a temperature of 25 °C and a relative humidity of 75%. The weight of the cup was recorded at intervals of 4 h for a period of 24 h. The WVP was calculated by analyzing the weight difference of the cups during the specified time intervals. The water contact angles of the composite films were measured using a contact angle meter (OSA100C, Ningbo New Boundary Scientific Instruments Co., Ltd., Ningbo, China).

### 2.7. Mechanical Properties

A texture analyzer (TA. XT Plus, Stable Micro Systems Ltd., Godalming, UK) was employed to evaluate film strips measuring 50 mm × 10 mm at stretching rate of 1.0 mm/s, recording the changes in relevant indices during the tensile process of the samples. The tensile strength (TS) and elongation at break (EB) of the films were calculated according to our previously established research method [20].

### 2.8. Passion Fruit Coating Treatment

The passion fruits used in this study underwent surface cleaning and were subsequently air-dried. Each group (10) of fruits was immersed in the film-forming solution for 30 s and air-dried at room temperature for 3 h. Following that, the fruits were immersed in a 2% calcium chloride solution for 30 s to facilitate cross-linking. After another round of air-drying at room temperature, the fruits were placed in plastic baskets and stored at room temperature. In the control group, the fruits were solely immersed in a 2% calcium chloride solution for 30 s. The weight loss and shrinkage index of the fruits were measured daily. The calculation method for the fruit shrinkage index was based on a previous study conducted by Zhang et al. [21].

### 2.9. Statistical Analysis

Each measurement was performed in triplicate, and the results were expressed as mean ± standard deviation. Statistical analysis was conducted using one-way analysis of variance (ANOVA) followed by Duncan’s multiple range test to determine significant differences (*p* < 0.05) between the samples.

## 3. Results and Discussion

### 3.1. Color and Opacity of SA Film

**Figure 1:**
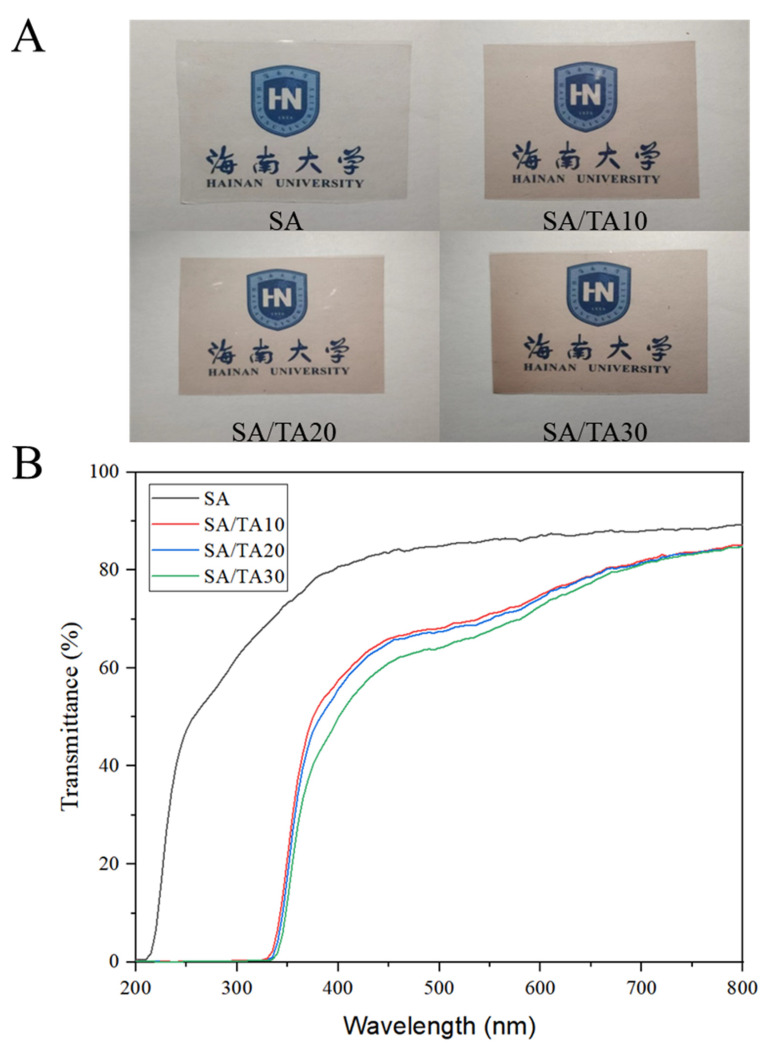
Pictures (**A**) and UV scan spectra (**B**) of SA and SA films doped with different concentrations of tannic acid. SA: sodium alginate; TA: tannic acid.

The appearance and surface morphology of food products play a crucial role in consumer evaluation and purchase decisions. The transparency of food packaging is an important factor in assessing its practicality. However, the exposure of food to ultraviolet (UV) light causes the degradation of nutrients, which in turn leads to the deterioration of the food in a short amount of time [22]. Therefore, it is not only necessary to consider the light transmittance of food packaging films in the visible spectrum but also to improve their UV barrier performance. Figure 1A shows images of SA films and SA films incorporated with different concentrations of TA. The neat SA films exhibited the highest visual transparency, while the incorporation of TA resulted in a gradual deepening of color, giving the films a lavender hue. However, there was no significant difference in color among the SA films with 10%, 20%, and 30% TA, indicating that the addition of TA did not significantly influence the perceived appearance of the food. The L*, a*, and b* values of the SA films exhibited significant changes with the addition of TA, as shown in Table 1. The neat SA films had the highest L* value, whereas the incorporation of tannic acid significantly decreased the L* value. Notably, the L* values of SA films containing 20% and 30% TA were significantly higher than those of SA films with 10% TA. This may be attributed to the reduced proportion of SA in the films as SA concentration increased, resulting in decreased brightness. However, TA formed complexes with SA in the films as TA concentration increased, thereby increasing their brightness. Additionally, the lower -b value and higher a value of the SA films indicated a transparent and bright color, while TA exhibited a brownish-yellow hue at room temperature. Consequently, the incorporation of TA increased the greenness and yellowness of the films. Importantly, although the addition of TA caused the color to be more yellow-green, there was no significant difference between SA membranes with different TA concentrations. This is primarily due to the limited variation in color caused by TA, which maintains good translucency. The total color difference value ΔE indicated that the color change in SA films incorporated with 30% TA compared to neat SA films was below 5, which is considered to have minimal color change, according to Liu et al. [23]. Hence, the incorporation of TA has a minimal impact on the color of SA films while maintaining good visual transparency, making them suitable for various food preservation and storage applications.

In addition, Figure 1B shows the UV-Vis spectral analysis of pure SA films and SA films with different concentrations of tannic acid. The pure SA films exhibited the lowest opacity (1.23 ± 0.07%), whereas the SA film with 10% TA had an opacity of 2.65 ± 0.17%, indicating that the incorporation of tannic acid significantly reduced the transparency of the SA film. Similarly, in another study, the incorporation of TA into Poly Lactide-Poly (Butylene Adipate-Co-Terephthalate) Blend films led to a decrease in transparency from 73.38 ± 0.15% to 58.41 ± 0.22% [24]. However, the opacity of SA films slightly decreased with increasing TA concentration, reaching 2.26 ± 0.11% opacity when blended with 30% TA. This may be attributed to the increase in film thickness resulting from TA incorporation. Furthermore, pure SA films exhibited the highest transmittance at UV 280 nm (58.83%), while the TA-doped SA films showed no transmittance at 280 nm due to the strong UV absorption capability of TA. Similarly, Roy et al. [25] found that TA contains benzene ring and carboxyl functional groups, which have strong UV absorption ability. When TA was added to chitosan films, it could almost completely block the penetration of UV rays, thus providing chitosan films with excellent UV protection properties. It is worth noting that the UV transmittance of SA films incorporated with different TA concentrations (10–30%) was similar. Although the transparency of SA films with 10–30% TA was significantly reduced compared to pure SA films, they still exhibited over 70% transparency in the visible range (400–800 nm), which is satisfactory for consumer preference in terms of appearance. Therefore, the uniform dispersion of TA in SA films can improve their UV-blocking performance while maintaining good transparency.

### 3.2. SEM Morphology of SA Films

**Figure 2:**
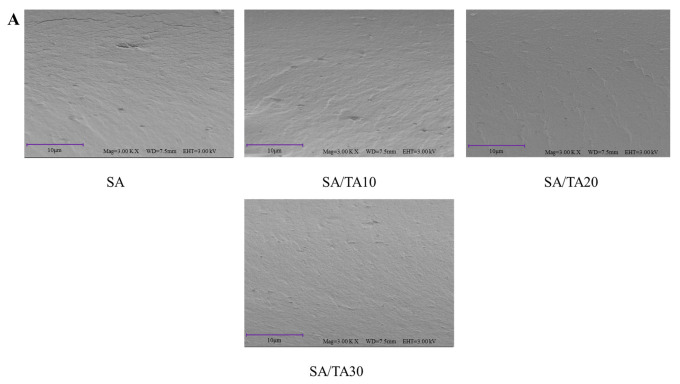

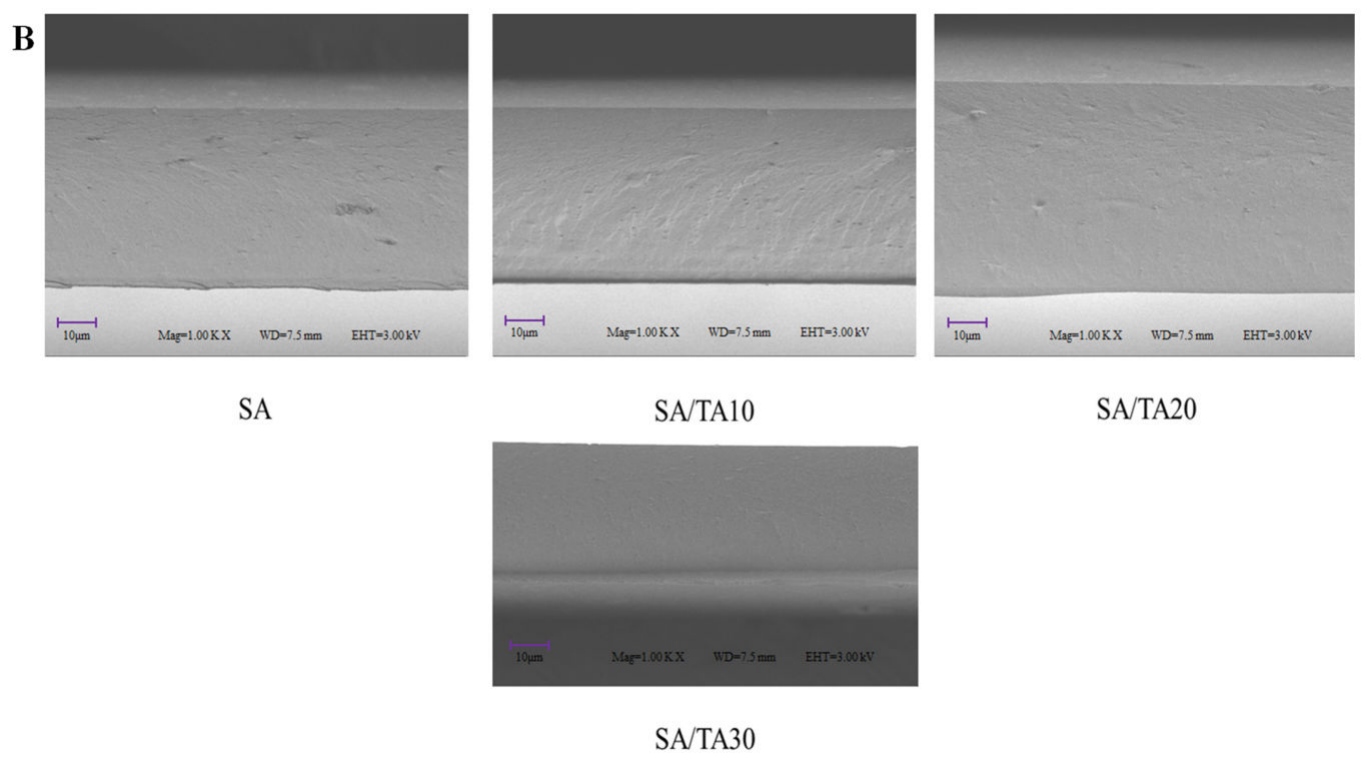
Surface (**A**) and cross-section SEM images (**B**) of SA, SA/TA10, SA/TA20, and SA/TA30 composite films. The magnifications of the surface and cross-section were 3000× and 1000×, respectively. SA: sodium alginate; TA: tannic acid.

The SEM images in Figure 2 illustrate the surface and cross-sectional morphology of neat SA films and SA films incorporated with 10–30% TA. Neat SA films displayed raised small grains and tiny folds on the surface. The presence of rapid gelation of alginate cross-linked with calcium ions (Ca^2+^) may contribute to the formation of an inhomogeneous structure [11]. TA has been reported to function as a bridging agent, enhancing the performance of calcium–alginate nanoparticles [26]. With the addition of TA, the presence of small grains on the surface of SA films decreased, particularly in the SA films containing 20% TA, where a smooth and flat surface was observed. This indicates that TA has a positive interaction with SA, resulting in a continuous and smooth surface morphology of the SA films, reducing the uneven gel structure formed by SA and Ca^2+^. It should be noted that white spots were observed in the SA films with 20% and 30% TA, which may be caused by the aggregation of TA, which is consistent with the observations made on chitosan films incorporating other polyphenol extracts [19]. In the cross-sectional images, holes and cracks were observed within the neat SA films, suggesting the presence of large voids between the SA molecules, resulting in a looser, net-like structure. However, with the incorporation of TA, the voids and cracks between the SA films were significantly reduced. The SA films containing 20% and 30% TA exhibited a uniform, smooth, and flat cross-section. This indicates that the addition of TA can effectively enhance the structure of SA films, making them more dense and compact, thus improving the physicochemical properties of the films. In a similar work, chitosan/starch films incorporating TA exhibited a denser and more continuous cross-sectional microstructure [27].

### 3.3. FTIR Analysis of the Chemical Structure of SA Films

**Figure 3:**
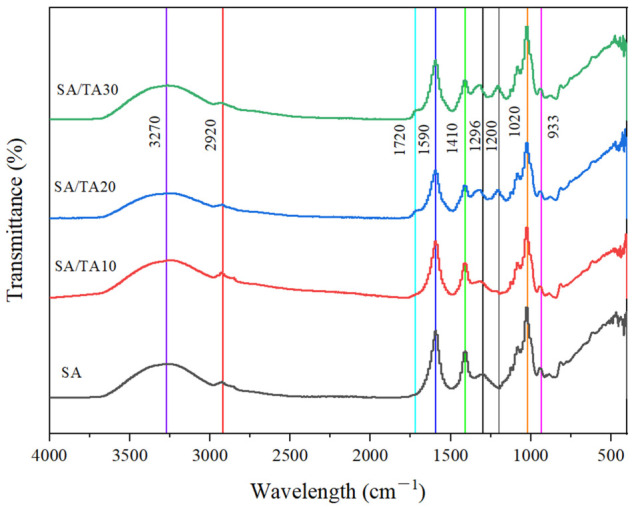
FTIR spectra of SA, SA/TA10, SA/TA20, and SA/TA30 films. SA: sodium alginate; TA: tannic acid.

The ATR-FTIR spectra in Figure 3 show the differences in the chemical structures of neat SA films and SA films incorporated with different TA concentrations. The characteristic peak at 3270 cm^−1^ of the SA film can be observed by the FTIR spectrum, resulting from the stretching vibrations of −OH bonds in the polysaccharide structure of SA [28]. Meanwhile, the peak at 2920 cm^−1^ in the neat SA film is derived from the -CH group band in SA [29]. Notably, sharp and intense absorption peaks were observed at both 1590 cm^−1^ and 1410 cm^−1^, which confirmed the asymmetric weak stretching vibration and symmetric strong stretching vibration of the carboxylate ion in SA [30]. And with the incorporation of TA, the peaks of the films in 1590 cm^−1^ and 1410 cm^−1^ gradually decreased, which may be attributed to the formation of hydrogen bonds between SA and TA. The increase of TA decreased the proportion of SA, which in turn resulted in a decrease of the peak in each band. Previous studies have reported that the hydroxyl groups of TA can form intermolecular hydrogen bonds with the carboxyl and hydroxyl groups of SA [2,31]. On the other hand, with the addition of TA, the SA films showed a strong absorption peak at 1200 cm^−1^, which was mainly attributed to the C-O polyphenol vibrational band in the TA structure, indicating the successful incorporation of TA into the SA films [32]. In addition, SA films were observed showing sharp and prominent peaks in the 1720 cm^−1^ band upon the addition of TA, which was mainly due to the stretching of the ester structure (C=O) in the TA [33]. Similarly, Zhang et al. [34] showed that the addition of TA to SA/Ca^2+^ hydrogels was able to observe the vibration of the C=O peak in the 1714 cm^−1^ spectrum, which confirmed that TA was successfully added to the SA matrix. In general, the position of the energy bands does not shift significantly after TA is doped into the sodium alginate film, but the intensity of the peaks changes considerably. This is mainly due to the hydrogen bonding interaction force between SA and TA.

### 3.4. XRD Crystal Structure Analysis

**Figure 4:**
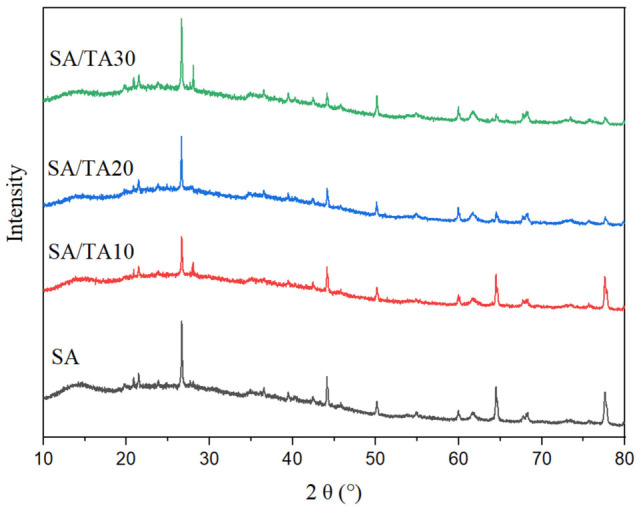
X-crystal diffraction analysis of SA, SA/TA10, SA/TA20, and SA/TA30 films. SA: sodium alginate; TA: tannic acid.

The XRD pattern analysis (Figure 4) shows that pure SA films exhibit a broad diffraction peak at 2θ = 20.9°, indicating a non-crystalline morphology. With the addition of TA, the pattern of SA films remained relatively unchanged, but the broad peak gradually became sharper. This indicated that the crystal structure of SA films is not significantly affected by the incorporation of TA. Furthermore, the peak at 2θ = 21.46° in the SA film shifts to 21.5° with the addition of TA, which can be attributed to the formation of hydrogen bonds between TA and SA. Additionally, the intensity peak at 2θ = 14.4° corresponding to SA gradually decreases as the TA concentration increases, indicating intermolecular interactions between TA and SA, which disrupt the crystal structure of SA films [35]. Similar findings have been reported by Larosa et al. [36], who loaded tannase on SA films and observed intermolecular interactions between tannase and alginate at 2θ = 13°, leading to the transformation of alginate from a crystalline state to an amorphous form. Therefore, the addition of TA converted the SA film into an amorphous structure, which facilitated the coordination interactions between SA and tannic acid.

### 3.5. Mechanical Properties of SA Film

**Figure 5:**
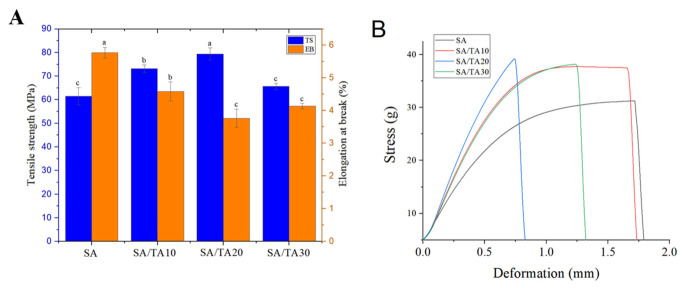
(**A**) Tensile strength (TS), elongation at break (EB), and (**B**) typical stress–strain curves of SA, SA/TA10, SA/TA20, and SA/TA30 films. SA: sodium alginate; TA: tannic acid. Moreover, a–c represent significant differences between the samples in each group.

The mechanical properties of films are often influenced by their thickness, as shown in Table 1, where the thickness of pure SA films was measured to be 45.7 ± 0.2 μm. With the addition of TA, the thickness of the SA films significantly increased, primarily due to the higher solid content resulting from the incorporation of TA. This observation is consistent with the findings of previous studies by Kamari et al. [32], which reported an increase in film thickness with the addition of TA in chitosan, gelatin, and methylcellulose films. Figure 5 presents the tensile strength (TS) and elongation at break (EB) of the SA films with different concentrations of TA, along with the corresponding stress–strain curves. In the context of food packaging, the TS and toughness of packaging materials are important factors to consider, given the processing, storage, and marketing of food products. The TA and EB serve as essential parameters for evaluating the mechanical properties of food packaging materials [17]. Previous research has shown that neat SA films exhibit moderate deformation plasticity, and the TS and EB of SA films can be enhanced by incorporating various active ingredients such as pentosidine. However, in this study, a significant increase in TS was observed in the SA films with the addition of different concentrations of TA. Compared to the neat SA films, the SA films containing 10% TA exhibited an approximately 15.88% increase in TS, accompanied by a 20.45% decrease in EB. With the incorporation of 20% TA, the SA films showed the highest TS of 79.45 ± 2.49, while the EB decreased to 3.76 ± 0.281. These findings align with the research conducted by Ruan et al. [37], indicating that an increase in TS tends to result in a decrease in EB. This phenomenon can be attributed to the ability of the polyphenolic structure of TA to form hydrogen bonds with SA, leading to the formation of a more compact network structure within the SA films, thereby significantly enhancing their TS. The SEM images of the films also confirm the presence of a dense structure in the SA/TA films, while the FTIR analysis further verifies the incorporation of TA and suggests the existence of strong interactions between TA and SA. However, when the concentration of TA incorporated into the SA films reaches 30%, a significant decrease in TS is observed, accompanied by a subsequent increase in EB. This phenomenon may be attributed to the high proportion of tannic acid in the SA films, leading to the aggregation of TA and causing structural disintegration between the polymers [38]. This observation is consistent with the SEM results, where small white spots resulting from TA aggregation are observed on the surface of the SA films containing 30% TA.

### 3.6. Moisture Content and Water Solubility of SA Film

The moisture content and water solubility of the neat SA films and SA films incorporated with different concentrations of TA are presented in Table 2. The moisture content of the neat SA films was measured to be 21.30 ± 0.34%. With the addition of TA, the moisture content of the SA films decreased as the TA concentration increased. This can be attributed to the highly hydrophilic nature of SA, which tends to adsorb water molecules, resulting in films with higher water content [39]. However, when TA is incorporated into the SA films, it forms a dense network structure with SA, filling the voids between the films and significantly reducing the water content of the SA films. Furthermore, SA exhibits high hydrophilicity, leading to a water solubility of 44.32 ± 2.92%. The incorporation of TA slightly decreased the water solubility of the SA films. The high hydrophilicity of TA contributes to increased water solubility when incorporated into SA films. Moreover, the addition of TA disrupts the original eggshell-like structure formed by the cross-linking of SA and Ca^2+^ in the SA films.

### 3.7. Water Vapor Permeability and Water Contact Angle

The water vapor permeability (WVP) coefficient is an important parameter for evaluating the moisture exchange between food packaged inside a film and the external environment, which affects food preservation and storage. The neat SA films have a high WVP value (0.205 ± 0.022 × 10^−10^ g m/m^2^·Pa·s) due to the hydrophilic nature of SA and its mesh structure that allows water molecules to pass through easily. Interestingly, SA films incorporated with 10–30% TA showed lower WVP values, with SA films containing 30% TA exhibiting the lowest WVP (0.156 ± 0.036 × 10^−10^ g m/m^2^·Pa·s). This finding is consistent with a study by Lee et al. [40]. Similarly, TA has been found to significantly reduce the WVP of gelatin films [15]. The formation of hydrogen bonds between TA and SA in the films creates a denser hydrogel network, reducing the transmission of water molecules. Additionally, the increase in film thickness and the presence of channel paths between the films result in a longer path for water molecules to traverse. Therefore, the incorporation of TA reduces the WVP of SA films, and the reduction is positively correlated with the TA concentration. Furthermore, the water contact angle of SA films was measured to evaluate their hydrophilicity. Neat SA films showed a water contact angle of 50.91 ± 1.07°, indicating moderate hydrophilicity. However, with the addition of TA, the water contact angle of SA films decreased significantly, reaching the lowest value (28.87 ± 0.22°) when 30% TA was incorporated. This decrease in water contact angle is attributed to the hydrophilic sites present in the TA structure [41]. Similarly, in a study by Lee et al. [40], the addition of TA to chitosan films resulted in a significant reduction in the water contact angle, which decreased further with increasing TA concentration. In general, an increase in the water content of the film is often accompanied by a decrease in the contact angle. Interestingly, in this study, both the water content and contact angle of the sodium alginate films showed a decrease with the addition of TA. This may be related to the interaction between TA and sodium alginate. Similarly, in another study, it was found that the addition of rose anthocyanins to the composite film of sodium alginate and carboxymethyl cellulose resulted in a simultaneous increase in the water content and water contact angle of the film [42]. The authors explained that the hydrophilicity of rose anthocyanins increased the water content of the film while, at the same time, rosmarinic acid increased the surface roughness of the film, leading to an increase in its hydrophobicity, which in turn increased the contact angle.

### 3.8. Thermal Stability of SA Films

**Figure 6:**
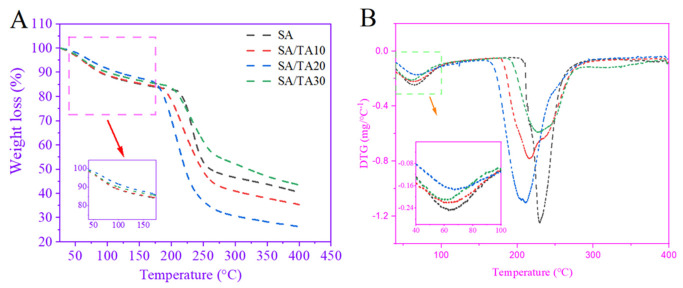
(**A**) TGA and (**B**) DTG curves of SA, SA/TA10, SA/TA20, and SA/TA30 films. SA: sodium alginate; TA: tannic acid.

The thermal stability of the films was evaluated using a thermogravimetric analyzer, and the thermal degradation curves (TG and DTG) of neat SA films and SA films incorporated with different concentrations of TA are presented in Figure 6. The first thermal degradation peak observed at 63 °C can be attributed to the evaporation of free water from the SA film [43]. It was observed that the SA films with 20% TA incorporation exhibited the lowest mass loss in the temperature range of 50–175 °C, while the neat SA films showed the highest mass loss. Theoretically, the lower the water content of the film, the lower the weight loss in thermogravimetric testing. However, we found that SA films containing 20% TA had significantly higher water content than SA films containing 30% TA but showed the lowest weight loss in water evaporation. This may be due to the fact that 20% TA was able to distribute more uniformly in the SA films and formed a dense hydrogen bonding network with the SA films, which restricted the flow of water molecules, while the agglomeration of 30% TA in the SA films reduced the binding of water molecules. The enhancement of mechanical properties and SEM micrographs mentioned above were also able to confirm this. Additionally, the neat SA films displayed a second peak of degradation at 229 °C, primarily associated with the decomposition of SA [44]. Interestingly, the SA films incorporated with TA showed a shift in the secondary degradation temperature toward lower temperatures, with the 20% TA blend demonstrating the lowest secondary degradation temperature (210 °C) (Figure 6B). Previous studies have indicated that polyphenolic compounds can undergo thermal conversion to generate thermosensitive radicals, leading to the densification of inter-polymeric network structures and a decrease in the degradation temperature of polymers [45]. Therefore, the presence of a polyphenol network in tannic acid may influence the degradation temperature of the films.

### 3.9. Passion Fruit Preservation Applications

**Figure 7:**
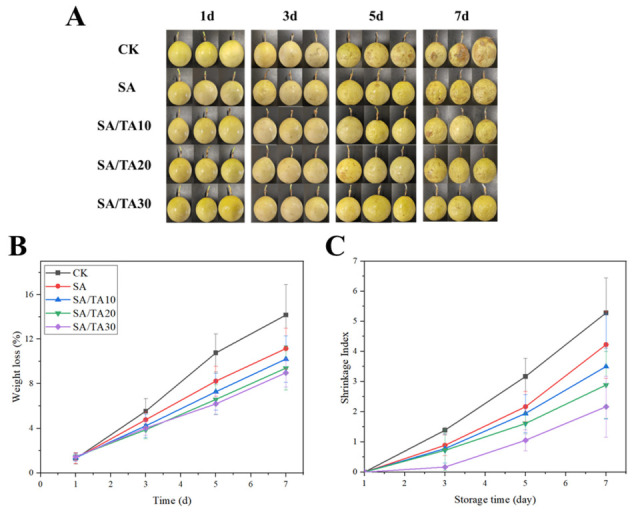
Appearance (**A**), weight loss (**B**), and wrinkle index (**C**) of passion fruit treated with SA and SA coating mixed with different concentrations of tannic acid for 7 days. SA: sodium alginate; TA: tannic acid.

Passion fruit is a highly sought-after fruit known for its good flavors and rich nutritional content, including anthocyanins and flavonoids [46]. In recent years, the demand for passion fruit has been increasing, leading to an expansion in its cultivation area in China [47]. However, passion fruit is a climacteric fruit, making it susceptible to dehydration, wrinkling, and microbial infestation during post-harvest storage, which significantly impacts its transportation and marketability. As depicted in Figure 7A, untreated passion fruit exhibited surface wrinkling and a deepened color after 3 days of storage. In contrast, passion fruit treated with SA coatings containing TA maintained a smooth surface, with only slight color changes observed in the passion fruit treated with SA coatings containing 10% TA. The control group displayed evident wrinkling and rotting on the 5th day, leading to complete spoilage on the 7th day. In contrast, passion fruit coated with SA coatings containing 10–30% TA exhibited varying degrees of wrinkling on the 5th day, which intensified on the 7th day. Passion fruit coated with SA coating containing 30% TA demonstrated minimal decay, although some wrinkling was observed. These findings highlight the superior freshness preservation of SA coatings containing 30% TA, which significantly delayed passion fruit decay. In addition, passion fruit is a respiratory leapfrog fruit with rapid transpiration and nutrient loss, resulting in water loss, crumpling, and rotting deterioration [48]. Therefore, we monitored the weight loss and wrinkle index of passion fruit for 7 days. As shown in Figure 7B, the weight loss of untreated passion fruit continuously increased during storage, reaching 14.16% on the 7th day. In contrast, passion fruit coated with SA coatings containing 10–30% TA exhibited weight loss rates below 11%, with the lowest weight loss (8.98%) observed in the passion fruit coated with SA coating containing 30% tannic acid. This indicates that passion fruit treated with SA coating containing 30% TA exhibited the best freshness preservation. The loose structure of the SA coating alone is not highly effective in reducing weight loss, but the incorporation of TA enabled the formation of a dense network structure within the SA coating, significantly enhancing its ability to retard weight loss in passion fruit. Additionally, TA possesses strong antioxidant and UV-blocking properties, which slow down the impact of external light and oxygen, thereby reducing the decomposition of surface substances and nutrients in passion fruit and maintaining its overall quality. In addition, the wrinkling index of passion fruit is shown in Figure 7C. The untreated passion fruit showed the fastest increase in the wrinkling index and was completely wrinkled on the 7th day. In contrast, SA-coated passion fruit demonstrated significantly lower wrinkling indices compared to the control group, albeit still exhibiting severe wrinkling. However, the incorporation of TA into the SA coating resulted in a significant reduction in the wrinkling index of passion fruit, with the lowest index observed in passion fruit coated with SA coating containing 30% TA. These findings align with previous studies that have demonstrated the effectiveness of edible coating treatments containing TA in preserving the quality of postharvest guava, mango, and passion fruit [49,50,51].

## 4. Conclusions

In this work, SA films with different TA concentrations (10%, 20%, and 30% *w*/*w*) were successfully prepared using the solution-casting evaporation method. The UV barrier properties, mechanical properties, and water vapor barrier properties of the SA films were determined, and the results showed that all three TA concentrations significantly enhanced the barrier and mechanical properties of the SA films. Among them, SA films incorporated with 20% TA exhibited the highest tensile strength, while SA films with 30% TA showed the lowest WVP. These improvements can be attributed to the formation of hydrogen bonds between the hydroxyl groups in TA and the carboxylate groups in the SA films. This was confirmed by SEM microscopic images and FTIR analyses, which showed structural changes and the presence of hydrogen bonds in the SA films. Additionally, compared to the control group, passion fruits treated with pure SA coatings and SA coatings containing tannic acid exhibited better delayed senescence effects for 7 days, with the effect becoming more pronounced with increasing TA concentration. Passion fruits treated with SA coatings containing 30% TA showed the lowest weight loss and crumpling index on the 7th day and maintained an excellent appearance. Therefore, incorporating 30% TA into SA films can be considered an effective strategy to delay senescence and preserve the quality of passion fruits. Overall, the results suggest that the addition of TA improves the barrier properties, mechanical properties, and thermal stability of SA films, making them suitable for applications in food coatings for fruit preservation.

## Figures and Tables

**Table 1:** Thickness and color parameters of SA, SA/TA10, SA/TA20, and SA/TA30 films.

Film	Thickness (μm)	L	a	b	ΔE	Opacity
SA	45.7 ± 0.2 ^c^	77.03 ± 0.62 ^a^	1.34 ± 0.12 ^c^	−2.04 ± 0.17 ^b^	14.03 ± 0.61 ^d^	1.23 ± 0.07 ^c^
SA-TA10%	50.9 ± 0.2 ^b^	61.44 ± 1.61 ^d^	6.34 ± 0.61 ^a^	2.25 ± 0.70 ^a^	23.88 ± 0.37 ^a^	2.65 ± 0.17 ^a^
SA-TA20%	53.8 ± 0.5 ^b^	64.56 ± 0.57 ^c^	5.32 ± 0.20 ^ab^	2.36 ± 0.40 ^a^	21.31 ± 0.93 ^b^	2.52 ± 0.11 ^a^
SA-TA30%	58.3 ± 0.7 ^a^	71.48 ± 1.82 ^b^	6.21 ± 0.69 ^b^	3.29 ± 1.11 ^a^	17.57 ± 0.99 ^c^	2.26 ± 0.11 ^b^

Values followed by the same letter in the same column were not significantly different according to Duncan’s Multiple Range Test (*p* < 0.05). Data are accompanied by standard errors of the means (*n* ≥ 3). SA: sodium alginate; TA: tannic acid. Moreover, a–d represent significant differences between the samples in each group.

**Table 2:** Moisture content, water solubility, water contact angle, and water vapor permeability of SA, SA/TA10, SA/TA20, and SA/TA30 films.

Types of Film	Moisture Content (%)	Water Solubility (%)	Water Contact Angle (°)	WVP (×10^−10^ g m/m^2^·Pa·s)
SA film	21.30 ± 0.34 ^a^	44.32 ± 2.92 ^c^	50.91 ± 1.07 ^a^	0.205 ± 0.022 ^a^
SA/TA10 film	20.02 ± 0.23 ^b^	55.11 ± 2.13 ^b^	32.40 ± 0.22 ^b^	0.185 ± 0.050 ^b^
SA/TA20 film	19.52 ± 0.42 ^b^	56.97 ± 0.89 ^b^	29.29 ± 0.68 ^c^	0.186 ± 0.019 ^b^
SA/TA30 film	18.35 ± 0.68 ^c^	64.17 ± 1.79 ^a^	28.87 ± 0.22 ^c^	0.156 ± 0.036 ^c^

Values followed by the same letter in the same column were not significantly different according to Duncan’s Multiple Range Test (*p* < 0.05). Data are accompanied by standard errors of the means (n ≥ 3). SA: sodium alginate; TA: tannic acid. Moreover, a–c represent significant differences between the samples in each group.

## Data Availability

Data is contained within the article.
